# Endothelial Cell-Specific Transcriptome Reveals Signature of Chronic Stress Related to Worse Outcome After Mild Transient Brain Ischemia in Mice

**DOI:** 10.1007/s12035-019-01822-3

**Published:** 2019-11-22

**Authors:** Stephanie Wegner, Ria Uhlemann, Valérie Boujon, Burcu Ersoy, Matthias Endres, Golo Kronenberg, Karen Gertz

**Affiliations:** 1 Klinik für Neurologie, Charité Campus Mitte, Charité—Universitätsmedizin Berlin, Charitéplatz 1, 10117 Berlin, Germany; 2 DZHK (German Center for Cardiovascular Research), Partner site Berlin, 10115 Berlin, Germany; 3 Deutsches Zentrum für Neurodegenerative Erkrankungen (DZNE), 10117 Berlin, Germany; 4 University of Leicester and Leicestershire Partnership NHS Trust, Leicester, UK

**Keywords:** Psychological stress, Depression, HPA axis, Stroke, Endothelium

## Abstract

Vascular mechanisms underlying the adverse effects that depression and stress-related mental disorders have on stroke outcome are only partially understood. Identifying the transcriptomic signature of chronic stress in endothelium harvested from the ischemic brain is an important step towards elucidating the biological processes involved. Here, we subjected male 129S6/SvEv mice to a 28-day model of chronic stress. The ischemic lesion was quantified after 30 min filamentous middle cerebral artery occlusion (MCAo) and 48 h reperfusion by T2-weighted MRI. RNA sequencing was used to profile transcriptomic changes in cerebrovascular endothelial cells (ECs) from the infarct. Mice subjected to the stress procedure displayed reduced weight gain, increased adrenal gland weight, and increased hypothalamic FKBP5 mRNA and protein expression. Chronic stress conferred increased lesion volume upon MCAo. Stress-exposed mice showed a higher number of differentially expressed genes between ECs isolated from the ipsilateral and contralateral hemisphere than control mice. The genes in question are enriched for roles in biological processes closely linked to endothelial proliferation and neoangiogenesis. MicroRNA-34a was associated with nine of the top 10 biological process Gene Ontology terms selectively enriched in ECs from stressed mice. Moreover, expression of mature miR-34a-5p and miR-34a-3p in ischemic brain tissue was positively related to infarct size and negatively related to sirtuin 1 (*Sirt1*) mRNA transcription. In conclusion, this study represents the first EC-specific transcriptomic analysis of chronic stress in brain ischemia. The stress signature uncovered relates to worse stroke outcome and is directly relevant to endothelial mechanisms in the pathogenesis of stroke.

**Electronic supplementary material:**

The online version of this article (10.1007/s12035-019-01822-3) contains supplementary material, which is available to authorized users.

## Introduction

Chronic psychosocial stress is increasingly recognized as a clinically meaningful cardio- and cerebrovascular risk factor [1–3]. Endothelial dysfunction may represent an important pathophysiological link whereby psychosocial stress confers increased vascular vulnerability [4, 5]. Even brief episodes of mental stress have been shown to elicit rapid and robust, albeit transient, effects on endothelial function [6, 7]. Studies in healthy human subjects indicate that, besides activation of the sympathetic nervous system, dysregulation of the hypothalamic-pituitary-adrenal axis contributes critically to stress-induced endothelial impairment [7–10].

Evidence from experimental animal research indicates that chronic stress increases infarct volume [11, 12]. By combining a mouse model of chronic stress over 4 weeks with a model of mild transient brain ischemia (i.e., 30 min middle cerebral artery occlusion [MCAo]), we previously identified that the adverse effects of stress on the ischemic brain involve the endothelium. Mechanistically, both glucocorticoid signaling and a stress-related increase in heart rate were found to cause endothelial dysfunction [13, 14].

Building on this evidence, the current study was undertaken to further characterize the neuroendocrine features of the four week stress model. After confirming that the stress procedure leads to increased lesion volume, we performed RNA sequencing to examine changes in gene expression due to prior stress exposure in ex vivo endothelia harvested from the middle cerebral artery (MCA) territory. We report a transcriptomic signature of chronic stress in MCAo-exposed endothelium and identify upregulation of miRNA-34a as a novel factor promoting ischemic brain injury.

## Materials and Methods

### Animals

All animal studies and experimental procedures were approved by the necessary official committees and conducted in compliance with the requirements set out in the European Communities Council Directive of November 24, 1986 (86/609/EEC) and the ARRIVE guidelines [15]. Male 129S6/SvEv mice raised under specific-pathogen-free (SPF) conditions were provided by the Forschungseinrichtungen für Experimentelle Medizin (FEM) of the Charité Universitätsmedizin Berlin. Young adult male mice (~ 9 ± 1 weeks old) weighing between 24 and 30 g were randomly used for experiments. Animals were maintained in a temperature (22 °C ± 2 °C) and humidity (55% ± 10%) controlled environment with a 12:12 h light-dark cycle and ad-libitum access to food and water.

### Stress Procedure

**Fig. 1:**
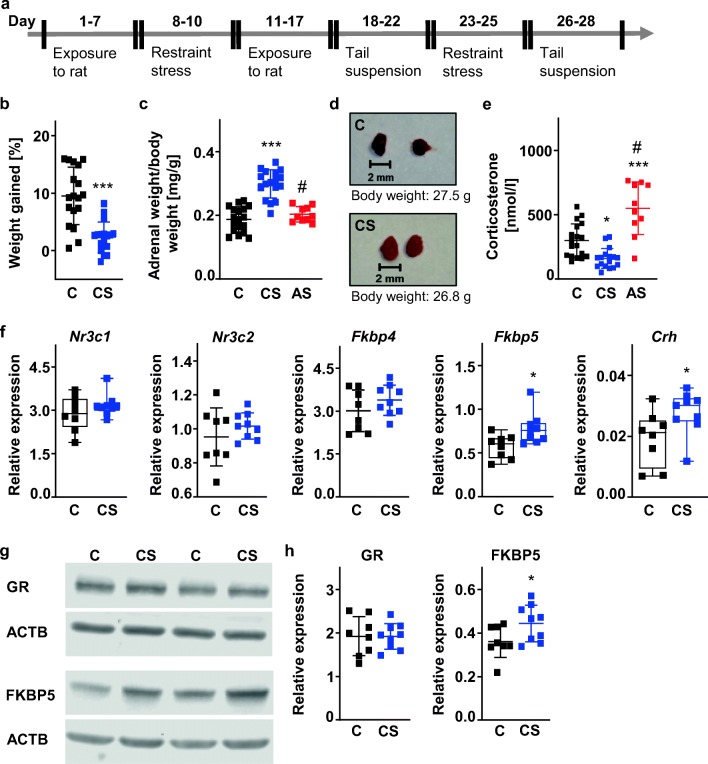
Characterization of the neuroendocrine effects of the chronic stress paradigm. **a** Schematic diagram of experimental design. **b** Body weight was recorded before the beginning and at the end of the chronic stress procedure. Chronically stressed mice gain less body weight than unstressed control animals. C: *n* = 18, CS: *n* = 17. Unpaired *t* test. *t* = 5.262, ***p < 0.001. **c**, **d** Chronic stress increases the weight of the adrenal glands. C: *n* = 18, CS: *n* = 17, AS: *n* = 10. One-way ANOVA F(2,42) = 40.14, *p* < 0.001 with Tukey’s multiple comparison test: ***p < 0.001 CS versus C, #*p* < 0.001 CS versus AS. **e** Corticosterone plasma levels were measured at the beginning of the light cycle. C: *n* = 18, CS: *n* = 17, AS: *n* = 10. One-way ANOVA F(2,42) = 26.20, *p* < 0.001 with Tukey’s multiple comparison test: *p < 0.05 CS versus C, ***p < 0.001 AS versus C, #*p* < 0.001 CS versus AS. **f** Hypothalamic mRNA transcription of genes associated with corticosteroid signaling. *Nr3c1*, nuclear receptor subfamily 3 group C member 1. *Nr3c2*, nuclear receptor subfamily 3 group C member 2. *Fkbp4*, FK506 binding protein 4. *Fkbp5*, FK506 binding protein 5. *Crh*, corticotropin releasing hormone. Relative mRNA expression is reported as the value normalized to tripeptidyl peptidase 2 (*Tpp2*) for each sample. C: *n* = 8, CS: *n* = 9. Mann-Whitney *U* test. *Nr3c1*: *U* = 27, *p* = 0.423. *Fkbp5: U* = 12, *p < 0.05. *Crh: U* = 14, *p < 0.05. Unpaired *t* test*. Nr3c2*: *t* = 1.016, *p* = 0.326. *Fkbp4*: *t* = 1.214, *p* = 0.244. **g** Representative Western blots of GR and FKBP5 protein expression in hypothalamic homogenates. GR, glucocorticoid receptor. FKBP5, FK506 binding protein 51. ACTB, β-actin. **h** Densitometric analysis. Values were normalized to β-actin. C: *n* = 8, CS: *n* = 9. Unpaired *t* test. GR: *t* = 0.029, *p* = 0.977. FKBP5: *t* = 2.173, *p < 0.05. C, unstressed control mice. CS, chronically stressed mice. AS, acutely stressed mice

A schematic of the experimental setup and timeline is given in Fig. 1a. Briefly, the stress procedure was adapted from previously published protocols [13, 14, 16].

#### Exposure to Rat

At the beginning of the dark phase, a single mouse was situated in a small cage with the following dimensions: height 140 mm, width 167 mm, length 252 mm. This small cage was then placed inside a larger rat cage (height 200 mm, width 375 mm, length 585 mm). A rat was introduced into the rat cage for 15 h (6 p.m. to 9 a.m.). A single session of exposure to rat was also used as the means to induce acute stress.

#### Restraint Stress

Animals were placed inside the restraining syringe (internal diameter 30 mm) for 1 h during the dark phase (7 p.m. to 8 p.m.).

#### Tail Suspension Stress

Mice were suspended by the tail approximately 80 cm above the ground for 6 min/day. The procedure started at 7 p.m.

### Induction of Cerebral Ischemia

The standard operating procedure ‘Middle cerebral artery occlusion in the mouse’ published by Dirnagl and members of the MCAO-SOP group was followed (for a more detailed description of the procedure please refer to http://precedings.nature.com/documents/3492/version/3/files/npre20123492-3.pdf). Briefly, mice were anaesthetized for induction with 1.5% isoflurane and maintained in 1.0% isoflurane in 69% N_2_O and 30% O_2_ using a vaporizer. Left MCAo was induced with an 8.0 nylon monofilament coated with a silicone resin/hardener mixture (Xantopren M Mucosa and Activator NF Optosil Xantopren, Heraeus Kulzer GmbH). The filament was introduced into the internal carotid artery up to the anterior cerebral artery. Thereby, the middle cerebral artery and anterior choroidal arteries were occluded. The filament was removed after 30 min to allow reperfusion.

### Magnetic Resonance Imaging

Successful MCAo was confirmed by magnetic resonance imaging (MRI) using a 7 Tesla rodent scanner (PharmaScan® 70/16, Bruker Corp.) and a 20-mm ^1^H-RF quadrature-volume resonator. A T2-weighted 2D turbo spin-echo sequence was used (imaging parameters TR/TE = 4200/36 ms, rare factor 8, 4 averages, 32 axial slices with a slice thickness of 0.5 mm, field of view of 2.56 × 2.56 cm, matrix size 256 × 256). Throughout the procedure, animals were anaesthetized with 1–2% isoflurane in 70% N_2_O and 30% O_2_. The respiratory rate was monitored with an MRI compatible small animal monitoring and gating system (SA Instruments, Inc.). Lesion volume was evaluated with Analyze 10.0 (AnalyzeDirect, Inc.) and corrected for edema according to a previously published protocol [17].

### Measurement of Adrenal Gland Weights and Corticosterone Plasma Levels

Corticosterone levels in plasma were determined by ELISA according to the manufacturer’s instructions (RE52211, IBL International GmbH). After sacrifice, the adrenal glands were dissected and weighed.

### RNA Isolation

Total RNA was extracted from hypothalamus and from ex vivo endothelial cells (ECs) using the NucleoSpin® XS Kit according to the manufacturer’s protocol (MACHEREY-NAGEL GmbH & Co. KG). RNA isolation from ECs was performed without the addition of carrier RNA.

### Quantitative Polymerase Chain Reactions

Synthesis of cDNA was performed with 300 U M-MLV reverse transcriptase (Promega Corp.), 20 U RNasin® ribonuclease inhibitor (Promega Corp.), 4.76 mM DTT (Promega Corp.), 1.43 mM PCR nucleotide mix (Promega Corp.), and 6.28 μM random primers (Roche Diagnostics GmbH). For PCR amplification, we used gene-specific primers (Table 1) and LightCycler® 480 SYBR Green I Master (Roche Diagnostics GmbH). Polymerase chain reaction conditions were as follows: preincubation 95 °C, 10 min; 95 °C, 10 s, primer-specific annealing temperature, 10 s, 72 °C, 15 s (45 cycles). Crossing points of amplified products were determined using the Second Derivative Maximum Method (LightCycler® 480 Version 1.5.0 SP4, Roche). Quantification of messenger RNA expression was relative to tripeptidyl peptidase (Tpp) 2 [18]. The specificity of polymerase chain reaction products was evaluated by melting curve analysis and electrophoresis on a 1.5% agarose gel.

**Table 1:** Oligonucleotide sequences used in quantitative real-time polymerase chain reactions

Gene	Sense	Antisense
*Crh*	CTA CCA AGG GAG GAG AAG AGA G	CTG CTC CGG CTG CAA GAA ATT C
*Fkbp4*	CGT GCT CAA GGT CAT CAA GAG AG	GTT GCC ACA GCA ATA TCC CAA GC
*Fkbp5*	GCA GGG TGA AGA TAT CAC TAC G	CCA ATG TCC CAG GCT TTG ATA AC
*Nr3c1*	GAC GTG TGG AAG CTG TAA AGT C	CCA GGT TCA TTC CAG CTT GAA G
*Nr3c2*	CAC ACG GTG ACC TGT CAT CTA G	CAT AGT GAC ACC CAG AAG CCT C
*Tpp2*	CTT CTA TCC AAA GGC TCT CAA GG	CTC TCC AGG TCT CAC CAT CAT G
*Sirt1*	CCA GAC CCT CAA GCC ATG TTT G	CTG CAA CCT GCT CCA AGG TAT C

### MiRNA Isolation and Quantification

Total RNA (including the microRNA fraction) was isolated from ischemic brain tissue using the Direct-zol^TM^ RNA MiniPrep Kit (Zymo Research Corp.). Tissue was homogenized with TRIzol^TM^ Reagent (Invitrogen^TM^) and TissueLyser LT (Qiagen) for 3 × 5 min at 50 Hz. Transcription of 500 ng total RNA into cDNA and real-time PCR was carried out with the miScript PCR Starter Kit (Qiagen). Custom miScript Primer Assays were used to detect miR-34a-5p, miR-34a-3p, and RNU6B (Qiagen). The level of miRNA expression in ischemic brain tissue of control mice and of chronically stressed mice was normalized to RNU6B (RNU6-2) using the 2^(-∆∆Ct)^ method [19]. Melting curve analysis and agarose gel electrophoresis confirmed specificity of polymerase chain reaction products.

### Western Blot

After sacrifice, brains were quickly removed, flash frozen in dry ice-cooled isopentane, and stored until further use. The hypothalamus was dissected on a cold plate (− 20 °C) according to Franklin and Paxinos (1997). Protein concentration was determined with the Pierce™ BCA Protein Assay Kit (ThermoFisher Scientific Inc.). Equal amounts of protein were loaded on 10% SDS-polyacrylamide gels and blotted onto Immobilon®-FL PVDF membranes (Merck KGaA). Near-infrared fluorescent signals were detected with the Odyssey® CLx Infrared Imaging System (LI-COR, Inc.). Densitometric quantification of band intensity was performed with the Image Studio™ Lite Software (LI-COR, Inc.). The following primary antibodies were used: rabbit anti-glucocorticoid receptor (#12041, Cell Signaling Technology, Inc.) 1:1000; rabbit anti-FKBP5 (#711292, ThermoFisher Scientific Inc.) 1:1000; mouse anti-beta actin (#ab8226, Abcam plc.) 1:2000. Secondary antibodies were used as follows: donkey anti-rabbit 800 (#925-32213, LI-COR, Inc.) 1:15000; donkey anti-mouse 680 (#925-32213, LI-COR, Inc.) 1:15000.

### Ex vivo Isolation of Brain ECs

Control (C) and chronically stressed mice (CS) were perfused transcardially with 0.9% saline. Brains were quickly removed. The ipsilateral ischemic MCA territory, as well as the corresponding area in the contralateral hemisphere, was carefully dissected and placed in cold PBS (4 °C). Brain tissue of 3–5 animals was pooled. CD31+ cells were enriched from brain tissue using a MACS protocol combined with prior myelin removal (Miltenyi Biotec GmbH). To ensure a high purity of ECs, an additional FACS step was performed. Cells were stained with DAPI and antibodies against CD31, CD146, and CD45. Single cells that were CD45-/DAPI-/CD31+/CD146+ were separated from the remainder of the suspension using a BD FACSAria™ II flow cytometer. For FACS staining, the following antibodies were used: anti-mouse CD31-Alexa Fluor® 488 (#102514, BioLegend, Inc.); anti-mouse CD45-APC (#130-102-544, Miltenyi Biotec GmbH); anti-mouse CD146 (LSEC)-PE (#130-102-319, Miltenyi Biotec GmbH).

### RNA Sequencing of Ex vivo ECs

Assessment of RNA quality, cDNA synthesis, library preparation, sequencing, data pre-processing, alignment, and principal component analysis (PCA) was conducted by LGC Genomics GmbH (Berlin, Germany). Libraries were prepared with the Encore Rapid DR Multiplex system (NuGEN Technologies, Inc.) according to the manufacturer’s directions and sequenced with 150 bp paired-end reads on the Illumina® NextSeq^TM^ 500. Data pre-processing involved the following steps: demultiplexing of all libraries with the Illumina® bcl2fastq 2.17.1.14 software, clipping of sequencing adapter remnants from all raw reads, merging of forward and reverse reads using BBMerge 34.48, SMRT concatamer adapter detection and read splitting, filtering of poly-A reads, and filtering of rRNA sequences with riboPicker 0.4.3 [20]. RNA-seq data have been deposited at the Gene Expression Omnibus (GEO) [21] under GEO accession number GSE122345.

### Analysis of Differentially Expressed Genes

A total of 400 million reads were aligned to the reference genome (*Mus musculus*; GRCm38; Ensembl version 84) using STAR 2.4.1b [22]. Reads mapping to rRNA or tRNA regions were filtered post-alignment. Counting of STAR-aligned reads was conducted using htseq-count [23]. FPKM values (fragments per kilobase per million fragments mapped) and differentially expressed genes (DEGs) analyses were calculated with Cuffdiff 2.1.1 by LGC Genomics GmbH (Berlin, Germany). The raw *p* values from the statistical tests were adjusted for multiple testing by the Benjamini-Hochberg false discovery rate (FDR) method. Genes with an FDR-adjusted *p* value < 0.05 and fold change ≥ 2 or ≤ − 2 were considered to be DEGs. When a gene cannot be detected in samples derived from one side of the brain, the log2(fold change) yields a value of infinity. In those instances in which the log2(fold change) yielded infinity and the FDR-adjusted *p* value was < 0.05, the gene in question was still not regarded as differentially expressed because these genes also showed a very low mean count of < 4 on the other side of the brain (5 genes in the control mice [*Gm13755, Gm3191, Igkv4-58, Gm6064, 1700017I07Rik*]; 2 genes in the chronically stressed animals [*Gm24447, Gm11686*]). The Venn diagram (Fig. 3c) was created with a web-based tool [24]. The expression levels of different marker genes were visualized with heatmapper [25]. Specific markers for each cell population were collated from different publications [26–33].

## Gene Ontology Analysis

Gene ontology (GO) enrichment analysis was performed with g:Profiler [34; Ensembl version 91]. The size of functional categories was limited to between 5 and 5000 genes so as to reduce redundancy in GO annotation. The Benjamini-Hochberg false discovery rate (FDR) correction was calculated to adjust for multiple testing. GO annotations with an adjusted *p* value < 0.01 were considered to be significantly enriched. Finally, REVIGO was used to filter out redundant categories [35].

## Statistics

Except for the stress experiments, all procedures and analyses were performed in a blinded fashion. Results are presented as individual values and as mean ± SD or as boxplot with median and interquartile range as appropriate. Mice were excluded from this study either when MRI yielded evidence of intracerebral hemorrhage (C: 0%, CS: 3%) or failed to confirm successful MCAo (C: 19%, CS: 21%). Statistical analysis was performed using GraphPad Prism version 7 or 8 (GraphPad Software, Inc.). Normality testing was done using D’Agostino-Pearson omnibus test (*n* ≥ 8) or Shapiro-Wilk test (*n* < 8). Since the present study was designed as an exploratory investigation, we did not correct for multiple comparisons.

## Results

### Neuroendocrine Effects of the Chronic Stress Paradigm

A schematic diagram of the experimental design is given in Fig. 1a. Briefly, three different stressors (i.e., exposure to rat, restraint stress, and tail suspension stress) were applied in an alternating fashion over the course of the experiment. Chronically stressed mice (CS) gained significantly less weight than control animals (C), which were left unperturbed in their home cages (Fig. 1b). Adrenal weight was significantly increased in CS mice after completion of the stress paradigm (Fig. 1c, d). Acute stress, i.e., a single session of exposure to rat before sacrifice early on the following morning, resulted in significantly increased circulating corticosterone levels and no relevant change in adrenal gland weight relative to C mice (Fig. 1c, e). By contrast, CS mice showed significantly reduced corticosterone plasma concentrations relative to C mice at the end of the experiment (Fig. 1e). Next, we studied mRNA transcription of genes associated with corticosteroid signaling in the left hypothalamus (Fig. 1f). Expression of glucocorticoid receptor (*Nr3c1*) mRNA and expression of mineralocorticoid receptor (*Nr3c2*) mRNA did not differ significantly between C and CS mice. However, CS mice showed significantly increased mRNA transcription of *Fkbp5* and *Crh* (Fig. 1f). Further, Western blot analysis of homogenates prepared from right hypothalamus confirmed increased FKBP5 protein expression in CS mice (Fig. 1g, h).

### Chronic Stress Impacts the Brain’s Sensitivity to Ischemic Injury

**Fig. 2:**
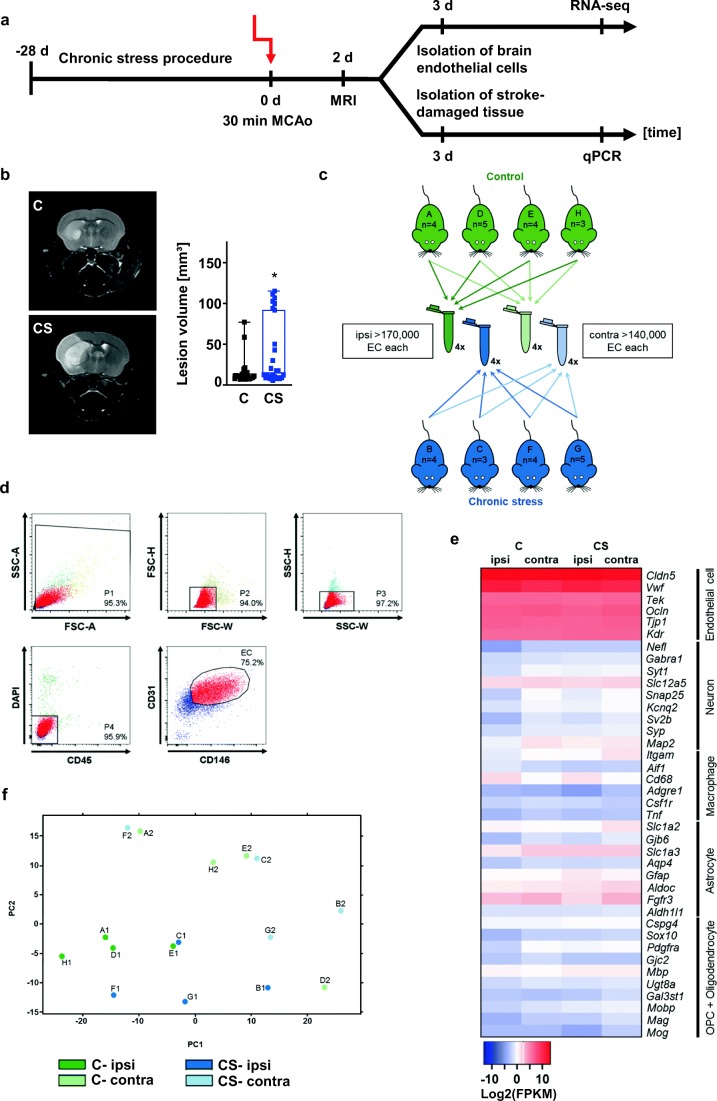
Chronic stress increases early lesion size after MCAo. **a** Experimental setup. Animals were either subjected to the 28-day stress procedure (CS) or were left unperturbed (C). On day 29, C and CS mice underwent 30 min MCAo/reperfusion. Successful MCAo was confirmed at 48 h by T2-weighted MRI. Mice were sacrificed 72 h after MCAo. **b** Chronic stress resulted in increased acute lesion sizes after 30 min MCAo. C: *n* = 25, CS: *n* = 26. Mann-Whitney *U* test. *U* = 213, *p < 0.05. **c**, **d** Endothelial cells (ECs) were harvested from the ipsilateral (ipsi; i.e., infarcted MCA territory) and contralateral (contra; i.e., corresponding area on the non-infarcted side) hemispheres of C and CS mice. Cells from 3–5 mice were pooled for each sample. **d** Gating strategy for flow cytometric sorting of MACS-purified CD31+ cells. **e** The heatmap confirms strong expression of endothelial marker genes in all samples. **f** Principal component analysis (PCA) plot of all RNA-seq data. The two-dimensional scatter plot represents the differential patterns of gene expression in ECs harvested from C and CS mice after 30 min MCAo/reperfusion. FPKM, fragments per kilobase per million fragments mapped

Next, we combined the chronic stress procedure with a model of mild transient brain ischemia (Fig. 2a). C and CS mice were used. Two days after 30 min MCAo/reperfusion, lesion size was measured in vivo using T2-weighted MRI. Lesion volume was found to be significantly increased in CS mice compared to C mice (Fig. 2b). Endothelial mechanisms have been implicated in linking psychological stress with stroke vulnerability [13, 14]. We therefore performed a gene expression analysis using ex vivo ECs harvested separately from the ischemic MCA territory and the corresponding area in the contralateral non-ischemic hemisphere (Fig. 2c). C and CS samples were pooled from groups of 3–5 mice (Fig. 2c). As illustrated in Fig. 2d, cells pre-enriched for CD31 were FACS purified to obtain CD45-/DAPI-/CD31+/CD146+ brain endothelia. An analysis of cell type-specific genes confirmed the purity of the FAC-sorted cells (Fig. 2e). Finally, a principal component analysis (PCA) performed on the RNA-seq data yielded two factors (Fig. 2f).

### A Transcriptomic Signature of Chronic Stress in MCAo-Exposed Endothelium

**Fig. 3:**
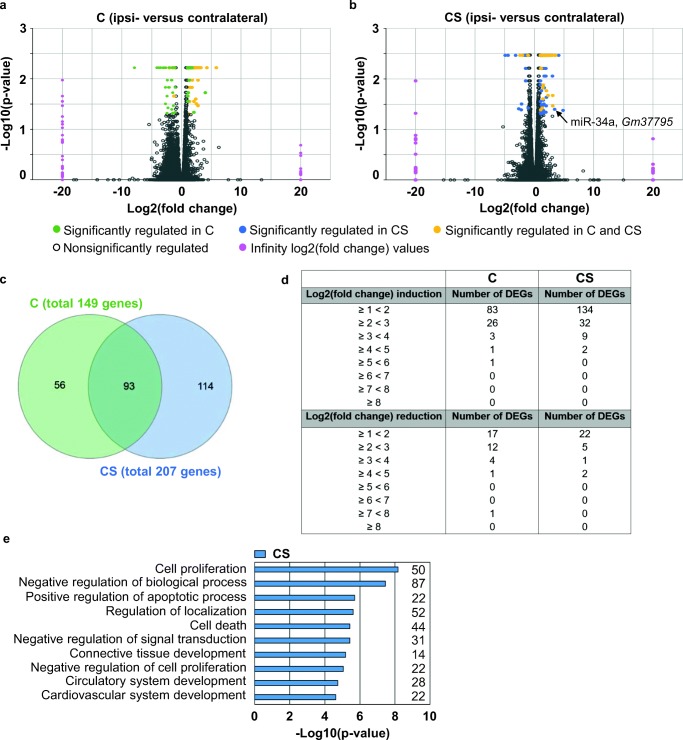
Transcriptomic analysis of brain ECs. ECs were harvested from C and CS mice 72 h after 30 min MCAo. Upregulation or downregulation of gene expression is relative to gene expression in endothelia harvested from the contralateral hemisphere. **a**, **b** Volcano plots of all genes quantified by RNA-seq. The negative log10 of the *p* value is plotted on the *y* axis. The *x* axis is the log2 of the fold change between endothelial gene expression in the ipsilateral and contralateral sides. **a** Gene expression in control (C) animals. **b** Gene expression in chronically stressed mice (CS). Green dots represent genes which are significantly upregulated or downregulated in C mice. Blue dots represent genes which are significantly upregulated or downregulated in CS mice. Yellow dots represent genes significantly regulated in both C and CS mice. Gray dots represent genes without significant regulation. The two vertical columns of pink dots in the chart are genes for which no expression was detected in ECs harvested from either the contralateral or the ipsilateral hemisphere, so log2(fold change) values were arbitrarily set to ± 20 (instead of ± infinity). Note that genes with a log2(fold change) value of infinity and FDR < 0.05 were not considered as differentially expressed due to low mean read counts. **c** Venn diagram showing the number and overlap of differentially regulated genes in ECs from C and CS mice. Green circle: endothelial gene expression in C mice; blue circle: endothelial gene expression in CS mice. **d** The table summarizes the number of differentially expressed genes (DEGs) in different log2(fold change) categories. **e** The top 10 biological process GO terms that were only detected in CS mice (see supplementary data for entire list)

Volcano plots show genes that were increased or decreased by more than two-fold (*p* < 0.05) in ipsilateral compared to contralateral endothelium (Fig. 3a, b). The number of differentially expressed genes (DEGs) was higher in endothelia from CS mice than in endothelia from C mice. Figure 3c summarizes the number of DEGs in C and CS mice as well as the intersection of genes regulated similarly in both experimental groups. A complete list of genes included in each category is provided in supplementary material ESM 2–4. Additionally, Fig. 3d summarizes the number of DEGs in different log2 (fold change) categories. RNA-seq did not yield differences in gene expression between ECs harvested from the contralateral hemisphere of C mice and ECs harvested from the contralateral hemisphere of CS mice.

Next, we performed a GO term analysis for the DEGs in each group. The annotation rates of the DEGs were 84.9% and 96.3% in ECs from C and CS mice, respectively. ESM 1a summarizes biological process GO terms that were simultaneously enriched in ECs from both C and CS mice (*p* < 0.01). Additional biological process GO terms emerged in CS mice. The top 10 of these terms are given in Fig. 3e. The biological processes in question are highly relevant to endothelial viability and function and highlight the effects of prior stress priming on the transcriptomic response to MCAo. The complete lists of GO terms enriched exclusively in C or CS mice are given in ESM 1b and 1c.

### Upregulation of MicroRNA-34a Promotes Ischemic Injury

MicroRNA-34a is increased by atheroprone oscillatory shear stress and promotes endothelial senescence [36, 37]. MiR-34a has previously been shown to repress SIRT1 expression [37, 38]. MiR-34a-5p and miR-34a-3p are the mature sequences of miR-34a.

MiR-34a was one of the transcripts found to be only differentially regulated in ECs from CS mice (Fig. 3b). Crucially, miR-34a was associated with nine of the top 10 biological process GO terms exclusively enriched in CS endothelium (Fig. 3e). We therefore investigated the relationship between expression of mature miR-34a-5p and miR-34a-3p in ischemic brain tissue and MRI lesion size.

**Fig. 4:**
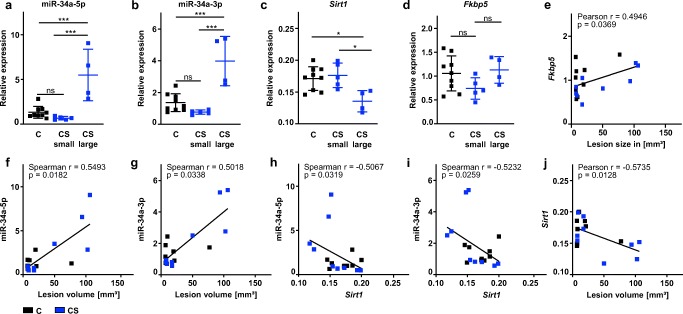
Expression of miR-34a microRNAs is positively related to lesion size and inversely related to *Sirt1* mRNA expression in ischemic whole brain. Mice were killed 72 h after 30 min MCAo/reperfusion. As described in the main text, CS mice were divided into two groups based on their infarct sizes—‘CS large’ group and ‘CS small’ group. Expression levels of miR-34a-5p, miR-34a-3p, *Sirt1*, and *Fkbp5* were assessed in ischemic whole brain tissue. MiR-34a values are expressed as fold change relative to RNU6B and one control mouse using the 2^(-∆∆Ct)^ method. Relative *Sirt1* and *Fkbp5* mRNA expression values were calculated using the ΔCT method corrected for primer efficiency and normalized to *Tpp2* as housekeeping gene. **a–d** C: *n* = 9, ‘CS small’ group: *n* = 5, ‘CS large’ group: *n* = 4. **e–j***N* = 9 mice per group. **a** One-way ANOVA F(2,15) = 16.28, *p* < 0.001 with Tukey’s multiple comparison test: ***p < 0.001. **b** One-way ANOVA F(2,15) = 19.82, *p* < 0.001 with Tukey’s multiple comparison test: ***p < 0.001. **c** One-way ANOVA F(2,15) = 6.575, *p* < 0.01 with Tukey’s multiple comparison test: *p < 0.05. **d** One-way ANOVA F(2,15) = 2.138, *p* = 0.152. **e–j** Pearson’s correlation coefficient or Spearman’s rank correlations were computed depending on whether data were normally distributed or not

There was considerable variance in infarct size among mice in the CS group, possibly reflecting partial resilience to the adverse effects of chronic stress in a subgroup of CS mice. For this reason, we divided CS mice into two groups based on their infarct sizes: (1) ‘CS large’ group with an infarct size above the median of the CS group, (2) ‘CS small’ group. Expression of miR-34a-5p and miR-34a-3p was significantly increased while *Sirt1* mRNA expression was significantly decreased in the ‘CS large’ group relative to both the ‘CS small’ group and C mice (Fig. 4a–c). Interestingly, mRNA transcription of *Fkbp5* observed from ischemic whole brain tissue did not differ significantly between experimental groups (Fig. 4d). However, we still found a moderate positive relationship between mRNA transcription of *Fkbp5* in ischemic brain tissue and infarct volume (Fig. 4e).

Expression of miR-34a-5p and miR-34a-3p was positively related to infarct size (Fig. 4f, g) and negatively related to *Sirt1* mRNA expression (Fig. 4h, i). Finally, transcription of *Sirt1* mRNA was negatively related to the size of the ischemic lesion (Fig. 4j).

## Discussion

While this study focuses chiefly on endothelial mechanisms, it is noted that the adverse effects of chronic stress are far-reaching and clearly not restricted to a single cell type in the brain. For example, the expression of neurotrophins in brain tissue is strongly impacted by stress and corticosteroids, which, in turn, may affect cellular plasticity and neuronal vulnerability to ischemia [39, 40]. With this being acknowledged, however, one key advantage to our reductionist approach is that it provides a new and hitherto barely considered angle on the complex pathways linking stress and stroke.

This study yielded the following key findings: (1) The chronic stress paradigm exerted robust effects at multiple levels, including, among others, reduced weight gain, increased adrenal gland weight, and increased hypothalamic FKBP5 mRNA and protein expression. (2) Using T2-weighted MR imaging, we replicate our earlier finding that the chronic stress paradigm sensitizes experimental animals to the effects of 30 min MCAo/reperfusion. (3) Next, we used RNA sequencing to profile gene expression changes in endothelia harvested from the ischemic brain as an effect of prior stress. We identified a distinct transcriptomic signature characterized by a higher number of DEGs in MCAo-exposed ECs derived from CS mice. Biological process GO terms enriched in ECs from CS mice are highly relevant to cell viability and function and include terms such as “cell proliferation,” “negative regulation of biological process,” “positive regulation of apoptotic process,” “cell death,” “negative regulation of signal transduction,” and “negative regulation of cell proliferation.” MicroRNA-34a is associated with nine of the top 10 biological process GO terms enriched in CS endothelium. (4) Expression of mature miR-34a-5p and miR-34a-3p in ischemic brain tissue was positively related to infarct size measured by MRI and negatively related to *Sirt1* mRNA expression.

Both reduced body weight [16, 41, 42] and increased body weight [43–45] have been observed with different rodent stress models. Factors that may explain differences between studies include the age, sex, and strain of the experimental animals, the kind of stressor used, and, importantly, the duration of the stressor [45, 46]. The chronic stress model was initially developed by Strekalova et al. with a view to producing a depression-like syndrome in male C57BL/6 mice [16]. For stroke experiments, our group adapted this model to 129S6/SvEv mice [13, 14], which, because of the nature of their cerebrovascular anatomy, lend themselves particularly well to transient intraluminal MCAo [47]. Our finding of reduced body weight in CS mice relative to non-stressed controls is in accord with the initial report by Strekalova et al. [16]. In line with our earlier study [13], the present investigation also confirms dysregulation of the HPA axis in the form of increased adrenal glands in CS mice. Furthermore, we show here that, in our model, chronic stress leads to upregulation of both *Crh* mRNA and FKBP5 mRNA and protein in hypothalamus. FKBP5 mRNA and protein expression is induced upon glucocorticoid receptor stimulation, providing an intracellular negative feedback loop for glucocorticoid receptor activity [48]. Together, these results strongly point to depression-like HPA axis hyperactivity and glucocorticoid resistance in mice subjected to 4 weeks of chronic stress [49, 50]. Interestingly, measurements in morning plasma performed at the end of the stress regime revealed decreased corticosterone concentrations in CS mice. A similar result has previously been reported in a chronic social defeat paradigm [45]. As plasma was taken 1 day after the end of the stress procedure and the animals had been left undisturbed in their home cages during the preceding dark phase, reduced trough corticosterone levels in CS mice most likely reflect adaptive changes of the HPA axis to chronic stress [45].

In the current study, we used the MCAo model to apply a cell-specific transcriptomic approach to uncover stress signatures in cerebrovascular endothelium. In doing so, we focused on molecular and physiological endpoints along with early lesion size on MRI. To our knowledge, this is the first report investigating the effects of chronic psychological stress on the endothelial transcriptome. Expression profiling of cerebrovascular endothelium following MCAo has likewise not yet been reported in the literature.

After completion of the 4-week chronic stress procedure, experimental mice were subjected to 30 min MCAo/reperfusion and sacrificed after an interval of 72 h. Our results, therefore, reflect the enduring effects of prior stress on stroke outcome. We acknowledge that our study does not address the connection between chronic stress, depressive-like behaviors, and functional outcomes after transient brain ischemia. However, in agreement with earlier studies using histological evaluation of infarct size [12–14, 51], T2-weighted imaging at 48 h confirmed a stress-induced increase in lesion volume.

ECs were isolated from the ipsilateral and contralateral MCA territory using sequential rounds of MACS and FACS. First, ECs were enriched by CD31 magnetic beads. Then, progressive FACS gating was used to remove cellular aggregates and cellular debris, as well as CD31 expressing hematopoietic cells [52, 53]. Purity of ECs was corroborated by robust expression of endothelial cell-specific marker genes alongside the absence of marker genes for other brain cells. Interestingly, we did not detect significant differences in gene expression between ECs harvested from the contralateral hemispheres of C and CS mice. This most likely suggests that, in our experimental setup, the 3-day interval between stress and sacrifice was too long for the effects of psychological stress to persist on the transcriptomic level in otherwise unperturbed brain endothelium. Of course, the effects of the intervening MCAo also have to be taken into consideration as the procedure itself is a strong stressor and leads to elevations in circulating corticosterone [e.g., 54].

In this analysis, we focused on genes that were either increased or decreased by more than two-fold (*p* < 0.05) in ipsilateral compared to contralateral ECs. As can be seen from the Volcano plots in Fig. 3, relatively few transcripts fell into this category. Biological process GO terms enriched in both C and CS mice are summarized in ESM 1a and reflect the broad cellular effects of ischemia on ECs. As described above, more DEGs were detected in CS than in C mice. Further exploration of our genomic findings will prove useful in elucidating the adverse effects of stress on ECs in the pathogenesis of stroke. For example, the upregulation of *Angpt2* in CS mice may point to altered blood vessel growth in the ischemic brain as angiopoietin-2 has been shown to impair revascularization after limb ischemia [55]. *Adamts9*, which was similarly upregulated in ipsilateral CS endothelium, has recently been identified as an endogenous angiogenesis inhibitor that operates cell-autonomously in ECs [56]. By contrast, mRNA expression of the brain-specific angiogenesis inhibitor 1 (*Adgrb1*) was not altered in CS endothelium, but strongly downregulated in ipsilateral ECs from C mice [57]. In the context of these observations, it is important to note that endothelial cell proliferation and angiogenesis are crucial regenerative mechanisms post-stroke [e.g., 58–61].

**Fig. 5:**
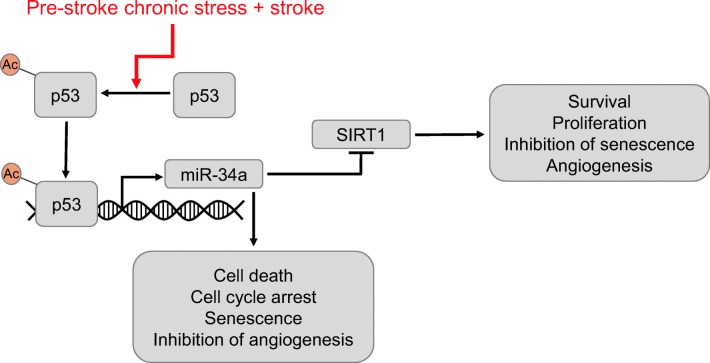
Upregulation of miR-34a in brain endothelia of CS mice after MCAo. The combination of ischemic injury and chronic stress results in increased transcriptional activity of p53, most likely through a posttranslational modification such as increased acetylation [38]. In consequence, miR-34a is upregulated, resulting in repression of SIRT1 and modulation of proliferation and apoptosis [37, 38]

Finally, microRNA-34a was selectively increased in CS endothelium from the ischemic brain. This finding is highly significant in the context of a confluence of research pointing to the importance of vascular mechanisms and regenerative angiogenesis for stroke outcome [58, 62, 63]. We speculate that pre-stroke stress in tandem with stroke injury enhances transcriptional activity of p53 most likely through its acetylation [38]. In turn, acetylated p53 upregulates miR-34a, thereby repressing SIRT1 and modulating proliferation and apoptosis [37, 38]. A schema of the putative underlying miR-34a pathway in brain endothelia is presented in Fig. 5.

There is ample evidence in the literature to indicate that changes in miR-34a and miR-34a target genes such as *Sirt1* will directly impact ischemic injury and, hence, infarct size. MiR-34a has been identified as a key driver of cardiovascular senescence [37, 64]. Moreover, anti-miR34a treatment following experimental myocardial infarction (MI) via coronary artery ligation significantly improved cardiac function post-MI [65]. Expression of miR-34a is elevated in senescent human umbilical cord vein endothelial cells as well as in heart and spleen of older mice [37]. MiR-34a induces G1 arrest, thereby blocking endothelial cell proliferation [37]. Conversely, inhibition of miR-34a by antisense oligonucleotides has been shown to increase capillary density in a mouse model of myocardial infarction, highlighting the favorable effects of inhibition of miR-34a on cardiac endothelium in the ischemic border zone [64]. Finally, SIRT1 is expressed by vascular endothelium in brain and has been shown to mediate cerebrovascular protection by facilitating NO-dependent vascular relaxation in a murine model of cerebral hypoperfusion induced by bilateral common carotid artery stenosis [66]. A growing body of evidence suggests that miRNAs may be released into the bloodstream or cerebrospinal fluid (CSF), making them attractive candidates for biomarker development. It is, therefore, especially notable that exciting new research has reported increased miR-34a-5p concentrations in blood and CSF of patients suffering from major depression [67]. Due to limited EC numbers, 3 to 5 samples per experimental condition had to be pooled in the current study. For this reason, it was not possible to directly correlate endothelial miR-34a expression to infarct size. However, in a subsequent experiment, we examined the relationship between MRI lesion size and expression of mature miR-34a-5p and miR-34a-3p in ischemic whole brain tissue. SIRT1 expression has been shown to be repressed by miR-34a [38]. Our analysis shows that expression of miR-34a-5p and miR-34a-3p was positively related to infarct size whereas *Sirt1* transcription was negatively related to infarct size. An obvious caveat to this finding is that miR-34a may be expressed by neurons as well as by other non-neuronal cells besides endothelia [68, 69].

In conclusion, this study identifies a transcriptomic signature of chronic stress in endothelia harvested from the ischemic murine brain. This stress signature relates to worse stroke outcome and is directly relevant to endothelial mechanisms in the pathogenesis of stroke.

## Electronic supplementary material

ESM 1 GO enrichment analysis of differentially expressed genes**. a.** Complete list of biological process GO terms enriched in both groups. **b.** Complete list of biological process GO terms which were only found to be significantly enriched in CS samples. **c.** Complete list of biological process GO terms enriched exclusively in samples derived from C mice. C, Control. CS, Chronic stress (PDF 105 kb)

ESM 2 DEGs that emerged in ECs from both control (C) and chronically stressed (CS) mice (PDF 64 kb)

ESM 3 DEGs that were only detected in ECs from C mice (PDF 30 kb)

ESM 4 DEGs that were only detected in ECs from CS mice (PDF 57 kb)
