# Supplementary figures and images for: Endothelial Cell-Specific Transcriptome Reveals Signature of Chronic Stress Related to Worse Outcome After Mild Transient Brain Ischemia in Mice
Source: Mol Neurobiol. 2019 Nov 22;57(3):1446–58. doi: 10.1007/s12035-019-01822-3 (PMC7060977; doi:10.1007/s12035-019-01822-3)

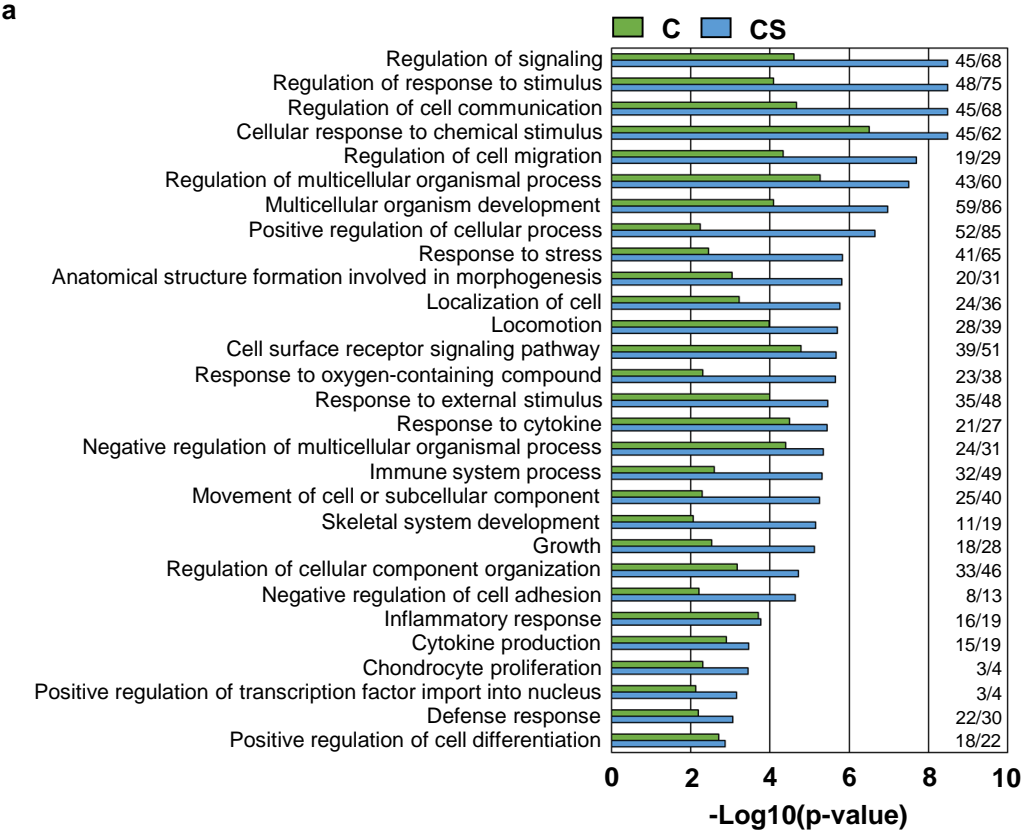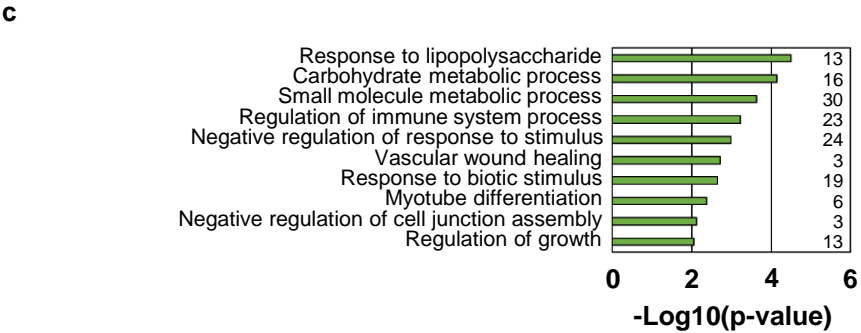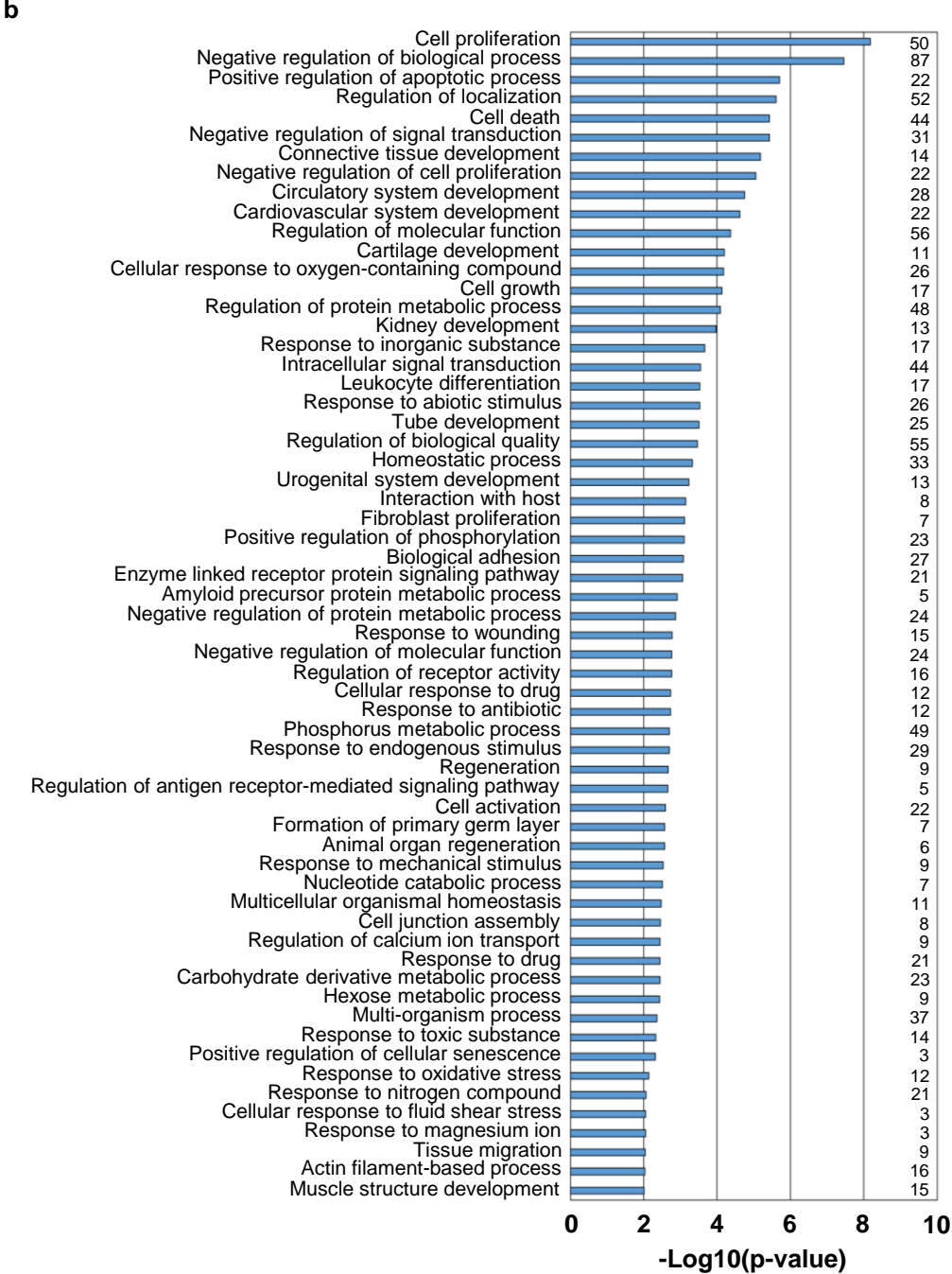

Supplement: Supplementary file 1 — GO enrichment analysis of differentially expressed genes. a. Complete list of biological process GO terms enriched in both groups. b. Complete list of biological process GO terms which were only found to be significantly enriched in CS samples. c. Complete list of biological process GO terms enriched exclusively in samples derived from C mice. C, Control. CS, Chronic stress (PDF 105 kb) [file 12035_2019_1822_MOESM1_ESM.pdf]
