# Supplementary material for: Endothelial Cell-Specific Transcriptome Reveals Signature of Chronic Stress Related to Worse Outcome After Mild Transient Brain Ischemia in Mice
Source: Mol Neurobiol. 2019 Nov 22;57(3):1446–58. doi: 10.1007/s12035-019-01822-3 (PMC7060977; doi:10.1007/s12035-019-01822-3)
Supplement: Supplementary file 2 — DEGs that emerged in ECs from both control (C) and chronically stressed (CS) mice (PDF 64 kb) [file 12035_2019_1822_MOESM2_ESM.pdf]

ESM 2 DEGs that emerged in ECs from both control (C) and chronically stressed (CS) mice

No	Gene	Log2(fold change)_C	FDR_C	Log2(fold change)_CS	FDR_CS	DE
1	*Itih4*	5.85	0.006	2.30	0.021	Up
2	*AA467197*	4.26	0.006	3.58	0.003	Up
3	*Lrg1*	3.24	0.006	2.70	0.003	Up
4	*Apod*	3.07	0.006	2.02	0.003	Up
5	*Scgb3a1*	2.94	0.006	2.36	0.003	Up
6	*Pdlim1*	2.93	0.006	3.46	0.003	Up
7	*Fbp1*	2.81	0.006	3.11	0.003	Up
8	*Csf2rb*	2.78	0.006	2.56	0.003	Up
9	*Igfbp6*	2.78	0.011	3.10	0.021	Up
10	*4933407L21Rik*	2.75	0.034	3.09	0.034	Up
11	*Ctsw*	2.59	0.006	1.63	0.003	Up
12	*Dusp2*	2.58	0.006	2.15	0.003	Up
13	*Serpine1*	2.50	0.031	2.05	0.017	Up
14	*Dok3*	2.43	0.006	1.75	0.003	Up
15	*Chst1*	2.43	0.006	2.88	0.003	Up
16	*Gpr55*	2.38	0.025	1.98	0.003	Up
17	*Ace*	2.30	0.006	1.60	0.003	Up
18	*Upp1*	2.23	0.006	2.87	0.003	Up
19	*Atp8b1*	2.23	0.006	2.01	0.003	Up
20	*Rnf183*	2.23	0.006	2.60	0.003	Up
21	*Tmem173*	2.21	0.006	2.15	0.003	Up
22	*Tnfsf8*	2.21	0.006	2.10	0.003	Up
23	*Cpe*	2.16	0.006	1.48	0.003	Up
24	*Lgmn*	2.11	0.006	1.81	0.003	Up
25	*Igfbp4*	2.10	0.006	2.00	0.003	Up
26	*Oaf*	2.08	0.028	2.51	0.003	Up
27	*Bmp4*	2.05	0.006	2.01	0.003	Up
28	*Myc*	2.05	0.006	2.28	0.003	Up
29	*Trp53i11*	2.02	0.006	2.56	0.003	Up
30	*Cd14*	1.94	0.006	2.90	0.003	Up
31	*Best1*	1.92	0.006	1.81	0.003	Up
32	*Lao1*	1.88	0.006	1.56	0.003	Up
33	*Dbn1*	1.79	0.006	1.47	0.013	Up
34	*Aldh3b1*	1.79	0.006	1.92	0.003	Up
35	*Spred3*	1.79	0.011	1.43	0.025	Up
36	*Inhbb*	1.76	0.006	2.57	0.003	Up
37	*Ifitm1*	1.75	0.006	2.79	0.003	Up
38	*Mmp14*	1.74	0.006	1.45	0.015	Up
39	*Sphk1*	1.74	0.015	1.83	0.003	Up
40	*Fosl1*	1.71	0.006	2.55	0.003	Up
41	*B3gnt3*	1.67	0.006	1.20	0.006	Up
42	*Gpr182*	1.67	0.006	1.41	0.003	Up
43	*Plekho2*	1.67	0.006	1.56	0.003	Up
44	*Hcls1*	1.65	0.006	1.95	0.003	Up
45	*4930486L24Rik*	1.62	0.006	1.69	0.003	Up
46	*Ppp1r14b*	1.59	0.006	1.59	0.003	Up
47	*Gmpr*	1.59	0.006	1.62	0.003	Up
48	*Tubb6*	1.56	0.006	2.35	0.003	Up
49	*Plekho1*	1.56	0.006	1.07	0.041	Up
50	*Glipr2*	1.54	0.006	1.78	0.003	Up
51	*Tmem252*	1.54	0.006	2.16	0.003	Up
52	*Synpo*	1.53	0.006	1.68	0.003	Up
53	*Ctla2b*	1.50	0.006	1.57	0.003	Up
54	*Adgrg3*	1.49	0.006	1.05	0.006	Up
55	*Hid1*	1.48	0.006	1.20	0.003	Up
56	*Mcam*	1.47	0.006	1.25	0.003	Up
57	*Snai1*	1.43	0.015	1.34	0.037	Up
58	*Acp5*	1.41	0.006	1.35	0.003	Up
59	*Slc10a6*	1.40	0.006	1.99	0.003	Up

No	Gene	Log2(fold change)_C	FDR_C	Log2(fold change)_CS	FDR_CS	DE
**60**	*Itm2c*	1.38	0.006	1.41	0.003	Up
**61**	*Ecscr*	1.38	0.006	1.55	0.003	Up
**62**	*Mustn1*	1.38	0.006	1.30	0.003	Up
**63**	*Marcksl1*	1.36	0.006	1.89	0.003	Up
**64**	*Ch25h*	1.35	0.006	1.75	0.003	Up
**65**	*Xbp1*	1.34	0.006	1.17	0.003	Up
**66**	*C1qtnf6*	1.32	0.028	1.91	0.003	Up
**67**	*BC018473*	1.25	0.011	1.69	0.003	Up
**68**	*Uchl3*	1.22	0.006	1.22	0.003	Up
**69**	*Pde4b*	1.21	0.006	1.52	0.003	Up
**70**	*Smim3*	1.21	0.006	1.47	0.003	Up
**71**	*Prr5*	1.21	0.050	1.59	0.003	Up
**72**	*Zfp521*	1.19	0.006	1.06	0.003	Up
**73**	*Rplp0*	1.19	0.006	1.04	0.003	Up
**74**	*Dnah6*	1.19	0.006	1.32	0.003	Up
**75**	*Hilpda*	1.19	0.006	1.15	0.003	Up
**76**	*Htra3*	1.16	0.006	1.08	0.003	Up
**77**	*Gm9625*	1.16	0.006	1.14	0.003	Up
**78**	*Adm*	1.16	0.006	1.74	0.003	Up
**79**	*Gm8730*	1.16	0.006	1.08	0.003	Up
**80**	*Apln*	1.11	0.028	3.47	0.003	Up
**81**	*Eva1a*	1.10	0.006	1.11	0.003	Up
**82**	*Plp2*	1.08	0.006	1.22	0.003	Up
**83**	*Ptpre*	1.07	0.006	1.81	0.003	Up
**84**	*Slc16a3*	1.03	0.015	1.43	0.003	Up
**85**	*Arrdc3*	1.02	0.011	1.26	0.003	Up
**86**	*Cmtm3*	1.01	0.006	1.14	0.003	Up
**87**	*Tnfrsf1a*	1.01	0.006	1.00	0.003	Up
**88**	*Pawr*	1.01	0.022	1.05	0.003	Up
**89**	*Il18bp*	1.00	0.011	1.10	0.003	Up
**90**	*Nov*	-2.08	0.006	-2.40	0.003	Down
**91**	*Slc26a10*	-1.46	0.006	-1.10	0.003	Down
**92**	*Klf15*	-1.30	0.022	-2.01	0.003	Down
**93**	*Pi16*	-1.14	0.006	-1.02	0.003	Down

DE = differentially expressed
