# Supplementary material for: Endothelial Cell-Specific Transcriptome Reveals Signature of Chronic Stress Related to Worse Outcome After Mild Transient Brain Ischemia in Mice
Source: Mol Neurobiol. 2019 Nov 22;57(3):1446–58. doi: 10.1007/s12035-019-01822-3 (PMC7060977; doi:10.1007/s12035-019-01822-3)
Supplement: Supplementary file 3 — DEGs that were only detected in ECs from C mice (PDF 30 kb) [file 12035_2019_1822_MOESM3_ESM.pdf]

ESM 3 DEGs that were only detected in ECs from C mice

No	Gene	Log2(fold change)	FDR	DE
**1**	*Wisp2*	3.96	0.019	Up
**2**	*2810459M11Rik*	2.07	0.046	Up
**3**	*Csf2rb2*	1.78	0.006	Up
**4**	*Plxnb2*	1.55	0.006	Up
**5**	*Abca1*	1.49	0.006	Up
**6**	*Steap3*	1.37	0.006	Up
**7**	*Lbp*	1.25	0.011	Up
**8**	*Ptgfrn*	1.22	0.006	Up
**9**	*Il20rb*	1.21	0.006	Up
**10**	*Scn1b*	1.19	0.006	Up
**11**	*Gadd45b*	1.18	0.006	Up
**12**	*Itpr3*	1.14	0.006	Up
**13**	*2410006H16Rik*	1.13	0.019	Up
**14**	*Fam43a*	1.11	0.006	Up
**15**	*Bcr*	1.11	0.006	Up
**16**	*Nid1*	1.11	0.006	Up
**17**	*Slco2b1*	1.09	0.006	Up
**18**	*Il4ra*	1.07	0.006	Up
**19**	*Socs3*	1.06	0.006	Up
**20**	*Spns2*	1.04	0.028	Up
**21**	*Gm6472*	1.04	0.006	Up
**22**	*Gm15500*	1.03	0.022	Up
**23**	*Rps11*	1.02	0.006	Up
**24**	*Pdia5*	1.01	0.006	Up
**25**	*Chst7*	1.01	0.022	Up
**26**	*Adgrb1*	-7.92	0.006	Down
**27**	*Gm27357,Gm27627,Gm27882,Gm27998,Miat*	-4.14	0.006	Down
**28**	*Gm27718,Gm27913,Gm27957,Pvt1*	-3.92	0.006	Down
**29**	*DQ267100,Gm23508,Gm24895,Gm26922,Gm27350,Rian*	-3.61	0.006	Down
**30**	*Psd4*	-3.57	0.006	Down
**31**	*4833445I07Rik*	-3.15	0.006	Down
**32**	*Sema4a*	-2.89	0.006	Down
**33**	*Bcat1*	-2.72	0.031	Down
**34**	*Catsperg2*	-2.68	0.015	Down
**35**	*Jakmip1*	-2.67	0.006	Down
**36**	*Gm27875,Jpx*	-2.45	0.022	Down
**37**	*Lemd1*	-2.45	0.048	Down
**38**	*Trpm1*	-2.41	0.011	Down
**39**	*Pappa2*	-2.30	0.037	Down
**40**	*Gm16503*	-2.20	0.015	Down
**41**	*Elavl2*	-2.06	0.015	Down
**42**	*Agxt2,Gm21973,Prlr*	-2.04	0.006	Down
**43**	*Dlgap1*	-1.72	0.011	Down
**44**	*Map2*	-1.65	0.011	Down
**45**	*Gm13034*	-1.65	0.040	Down
**46**	*Ugt1a1,Ugt1a10,Ugt1a2,Ugt1a5,Ugt1a6a,Ugt1a6b,Ugt1a7c,Ugt1a8,Ugt1a9*	-1.63	0.006	Down
**47**	*Maf*	-1.50	0.048	Down
**48**	*Adam22*	-1.48	0.006	Down
**49**	*Arpp21*	-1.47	0.019	Down
**50**	*Meis2*	-1.46	0.048	Down
**51**	*Ubn2*	-1.46	0.006	Down
**52**	*Ptprd*	-1.36	0.034	Down
**53**	*Eif3j2*	-1.36	0.006	Down
**54**	*Acta2*	-1.08	0.015	Down
**55**	*Alpl*	-1.04	0.006	Down
**56**	*Gm9946*	-1.04	0.046	Down

DE = differentially expressed
