# Supplementary material for: Endothelial Cell-Specific Transcriptome Reveals Signature of Chronic Stress Related to Worse Outcome After Mild Transient Brain Ischemia in Mice
Source: Mol Neurobiol. 2019 Nov 22;57(3):1446–58. doi: 10.1007/s12035-019-01822-3 (PMC7060977; doi:10.1007/s12035-019-01822-3)
Supplement: Supplementary file 4 — DEGs that were only detected in ECs from CS mice (PDF 57 kb) [file 12035_2019_1822_MOESM4_ESM.pdf]

ESM 4 DEGs that were only detected in ECs from CS mice

No	Gene	Log2(fold change)	FDR	DE
1	*Gm5134*	4.83	0.042	Up
2	*Kcne3*	4.07	0.003	Up
3	*Gm37795,Mir34a*	3.40	0.040	Up
4	*Rnf225*	3.02	0.009	Up
5	*Ackr1,Cadm3*	3.00	0.003	Up
6	*Csf3*	2.77	0.003	Up
7	*Pgf*	2.77	0.003	Up
8	*Ctla2a*	2.22	0.003	Up
9	*Kif17,Sh2d5*	2.16	0.003	Up
10	*Fscn1*	2.11	0.003	Up
11	*Cda*	2.04	0.023	Up
12	*Mdfi*	2.04	0.003	Up
13	*Lyve1*	2.01	0.003	Up
14	*Fbp2*	1.98	0.034	Up
15	*Odc1*	1.96	0.003	Up
16	*Psors1c2*	1.94	0.006	Up
17	*Rab20*	1.94	0.003	Up
18	*Car13*	1.90	0.046	Up
19	*C1qtnf5,Mfrp*	1.90	0.009	Up
20	*Cxcl16*	1.71	0.003	Up
21	*Hmga1*	1.68	0.015	Up
22	*Plpp2*	1.66	0.003	Up
23	*Angpt2*	1.65	0.009	Up
24	*Kit*	1.65	0.003	Up
25	*Gm7993*	1.61	0.006	Up
26	*Lgals3*	1.61	0.003	Up
27	*Pycr1*	1.60	0.031	Up
28	*Mt2*	1.60	0.003	Up
29	*Acot7*	1.55	0.003	Up
30	*Adamts9*	1.50	0.047	Up
31	*Slc16a6*	1.49	0.042	Up
32	*Kcnj15*	1.48	0.026	Up
33	*Fkbp1a*	1.47	0.003	Up
34	*Itgb3*	1.46	0.006	Up
35	*Ccnd1*	1.45	0.003	Up
36	*Nt5e*	1.42	0.033	Up
37	*Frzb*	1.41	0.048	Up
38	*Osgin1*	1.36	0.003	Up
39	*Cd82*	1.32	0.003	Up
40	*Hmga1-rs1*	1.30	0.003	Up
41	*Gale*	1.30	0.003	Up
42	*Sdcbp2*	1.29	0.003	Up
43	*Spp1*	1.29	0.003	Up
44	*Kcnj8*	1.28	0.003	Up
45	*Tfpi2*	1.26	0.036	Up
46	*Mt1*	1.26	0.006	Up
47	*Meox1*	1.22	0.003	Up
48	*Tuba1c*	1.21	0.003	Up
49	*Tnfrsf10b*	1.21	0.003	Up
50	*Stx11*	1.21	0.003	Up
51	*Nectin2*	1.21	0.017	Up
52	*Litaf*	1.20	0.003	Up
53	*Plaur*	1.19	0.003	Up
54	*S100a6*	1.18	0.003	Up
55	*Lgals1*	1.18	0.039	Up
56	*Map1b*	1.17	0.003	Up
57	*Rnd1*	1.17	0.003	Up
58	*Crlf2*	1.17	0.003	Up
59	*Abi3*	1.16	0.033	Up

No	Gene	Log2(fold change)	FDR	DE
**60**	*Serpinh1*	1.15	0.003	Up
**61**	*Gm20489,Gm614,Il2rg*	1.13	0.003	Up
**62**	*Gm10282*	1.12	0.050	Up
**63**	*Ube2m*	1.11	0.003	Up
**64**	*Phlda1*	1.11	0.003	Up
**65**	*Emp1*	1.10	0.003	Up
**66**	*Eif4ebp1*	1.09	0.003	Up
**67**	*Nrarp*	1.08	0.003	Up
**68**	*Hmgn2*	1.07	0.006	Up
**69**	*Ifitm3*	1.07	0.003	Up
**70**	*Smad1*	1.06	0.003	Up
**71**	*Gapdh*	1.05	0.013	Up
**72**	*Kctd17*	1.05	0.003	Up
**73**	*Ubtd1*	1.05	0.003	Up
**74**	*Ppa1*	1.04	0.003	Up
**75**	*Nrgn*	1.03	0.046	Up
**76**	*Hmox1*	1.03	0.003	Up
**77**	*Pdia6*	1.03	0.003	Up
**78**	*Stc1*	1.03	0.013	Up
**79**	*Ctsb*	1.02	0.003	Up
**80**	*Timeless*	1.02	0.003	Up
**81**	*Tcf19*	1.02	0.003	Up
**82**	*Sh3bp5*	1.01	0.003	Up
**83**	*Sod3*	1.01	0.028	Up
**84**	*5031439G07Rik*	1.00	0.003	Up
**85**	*Mall*	1.00	0.003	Up
**86**	*Spry4*	1.00	0.003	Up
**87**	*Lrrc32*	1.00	0.003	Up
**88**	*Gm28045,Prnd,Prnp*	1.00	0.011	Up
**89**	*Rttn*	-4.91	0.003	Down
**90**	*Pm20d1*	-4.45	0.003	Down
**91**	*Gria2*	-3.19	0.003	Down
**92**	*Ptpn6*	-2.65	0.039	Down
**93**	*Wnt11*	-2.24	0.041	Down
**94**	*Plch2*	-2.18	0.031	Down
**95**	*Dock7*	-1.68	0.003	Down
**96**	*Doc2b*	-1.68	0.003	Down
**97**	*B4galnt1*	-1.48	0.006	Down
**98**	*Vwa3a*	-1.47	0.011	Down
**99**	*Fgfr3*	-1.46	0.003	Down
**100**	*Trp53inp1*	-1.43	0.003	Down
**101**	*Nr4a1*	-1.43	0.003	Down
**102**	*Exoc3l2*	-1.41	0.003	Down
**103**	*Tmcc2*	-1.33	0.003	Down
**104**	*Kank4*	-1.19	0.003	Down
**105**	*Lamc3*	-1.15	0.003	Down
**106**	*Acacb*	-1.13	0.003	Down
**107**	*Cyp26b1*	-1.11	0.003	Down
**108**	*Cytl1*	-1.10	0.003	Down
**109**	*Nog*	-1.09	0.003	Down
**110**	*Tprn*	-1.06	0.003	Down
**111**	*Mycl*	-1.03	0.037	Down
**112**	*Palm*	-1.02	0.003	Down
**113**	*Pmaip1*	-1.02	0.003	Down
**114**	*Trpv4*	-1.00	0.003	Down

DE = differentially expressed
